# Chromosomal Differentiation in Genetically Isolated Populations of the Marsh-Specialist *Crocidura suaveolens* (Mammalia: Soricidae)

**DOI:** 10.3390/genes11030270

**Published:** 2020-03-02

**Authors:** Francisca Garcia, Luis Biedma, Javier Calzada, Jacinto Román, Alberto Lozano, Francisco Cortés, José A. Godoy, Aurora Ruiz-Herrera

**Affiliations:** 1 Unitat de Cultius Cel.lulars (UCC), Universitat Autònoma de Barcelona, Campus UAB, 08193 Cerdanyola del Vallès, Spain; francisca.garcia@uab.cat (F.G.); francisco.cortes@uab.cat (F.C.); 2 Department of Integrated Sciences, Faculty of Experimental Sciences, University of Huelva, Avenida de las Fuerzas Armadas, S/N, 21007 Huelva, Spain; setebiedma@hotmail.com (L.B.); javier.calzada@dbasp.uhu.es (J.C.); 3 Department of Conservation Biology, Doñana Biological Station, CSIC, C. Americo Vespucio 26, 41092 Sevilla, Spain; jroman@ebd.csic.es; 4 Departament de Biologia Cel·lular, Fisiologia i Immunologia, Universitat Autònoma de Barcelona, Campus UAB, 08193 Cerdanyola del Vallès, Spain; alberto.lozanom@e-campus.uab.cat; 5 Genome Integrity and Instability Group, Institut de Biotecnologia i Biomedicina, Universitat Autònoma de Barcelona, 08193 Cerdanyola del Vallès, Spain; 6 Department of Integrative Ecology, Doñana Biological Station, CSIC, C. Americo Vespucio 26, 41092 Sevilla, Spain

**Keywords:** *Crocidura suaveolens*, shrews, habitat specialist, chromosomes, chromosomal evolution, B-chromosomes, chromosomal polymorphism, mtDNA

## Abstract

The genus *Crocidura* represents a remarkable model for the study of chromosome evolution. This is the case of the lesser white-toothed shrew (*Crocidura suaveolens*), a representative of the Palearctic group. Although continuously distributed from Siberia to Central Europe, *C. suaveolens* is a rare, habitat-specialist species in the southwesternmost limit of its distributional range, in the Gulf of Cádiz (Iberian Peninsula). In this area, *C. suaveolens* is restricted to genetically isolated populations associated to the tidal marches of five rivers (Guadiana, Piedras, Odiel, Tinto and Guadalquivir). This particular distributional range provides a unique opportunity to investigate whether genetic differentiation and habitat specialization was accompanied by chromosomal variation. In this context, the main objective of this study was to determinate the chromosomal characteristics of the habitat-specialist *C. suaveolens* in Southwestern Iberia, as a way to understand the evolutionary history of this species in the Iberian Peninsula. A total of 41 individuals from six different populations across the Gulf of Cádiz were collected and cytogenetically characterized. We detected four different karyotypes, with diploid numbers (2n) ranging from 2n = 40 to 2n = 43. Two of them (2n = 41 and 2n = 43) were characterized by the presence of B-chromosomes. The analysis of karyotype distribution across lineages and populations revealed an association between mtDNA population divergence and chromosomal differentiation. *C. suaveolens* populations in the Gulf of Cádiz provide a rare example of true karyotypic polymorphism potentially associated to genetic isolation and habitat specialization in which to investigate the evolutionary significance of chromosomal variation in mammals and their contribution to phenotypic and ecological divergence.

## 1. Introduction

Large-scale chromosomal changes, such as inversions, translocations, fusions and fissions, contribute to the reshuffling of genomes, thus providing new chromosomal forms on which natural selection can work. In this context, genome reshuffling has important evolutionary and ecological implications, since gene flow can be reduced within the reorganized regions in the heterokaryotype, thus affecting co-adapted genes locked within the rearrangement that, if advantageous, increase in frequency in natural populations (reviewed in [1]). In fact, evidence on the role of large-scale chromosomal changes in adaptation and diversification has been reported, especially in the case of inversions [2,3,4,5]. As for chromosomal fusions, however, empirical studies are limited to the house mouse (*Mus musculus domesticus*) and shrews (*Sorex araneus*), two mammalian systems where the presence of chromosomal fusions (either fixed or in polymorphic state within populations) are widespread [6,7,8,9,10,11,12,13]. Understanding the genetic and mechanistic basis of these processes will provide insights into how biodiversity originates and is maintained.

Shrews (family Soricidae) represent a clear example of chromosomal diversification within mammals, with diploid numbers ranging from 2n = 19 (*Blarina hylophaga*) to 2n = 68 (*Crocidura yankariensis*), showing both inter- and intra-specific chromosomal diversity [14]. Within Soricidae, the genus *Crocidura* represents a remarkable model where karyotypic variation correlates with phylogenetic relationships within the group. Initial studies based on nuclear and mitochondrial sequences (mtDNA) suggested a common ancestry of all *Crocidura* species [15,16], with a clear dichotomy between Afrotropical and Palearctic taxa, which display contrasting patterns of chromosomal differentiation [17]. With the exception of *C. luna* (2n = 28 or 36 [18]) Afrotropical species are characterized by high diploid numbers (from 2n = 42 to 2n = 68), 2n = 50 being the most common chromosomal form. Palearctic species, on the other hand, present a tendency for low diploid numbers (from 2n = 22 to 2n = 42, [17], with 2n = 40 as the predominant karyotype. It has been suggested that this bimodal distribution of diploid numbers within the genus *Crocidura* is probably the result of two contrasting tendencies in chromosomal evolution: (i) chromosomal fissions in Afrotropical species, and (ii) chromosomal fusions (tandem fusions centric fusions, and/or whole arm translocations) in Palearctic species [19].

**Figure 1:**
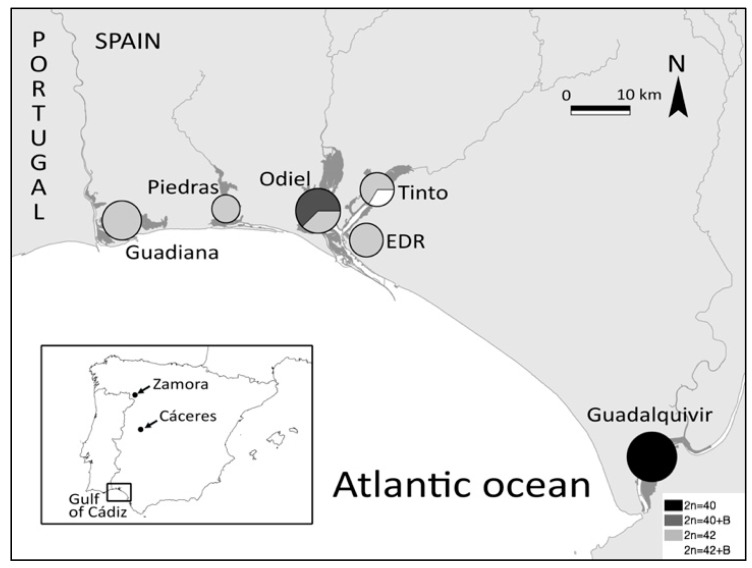
Study area and populations of *C. suaveolens* sampled in the tidal marsh (dark gray) of the Gulf of Cádiz, Southwestern Iberia. The individuals of Cáceres and Zamora are shown in the inset map. The sampling locations and diploid numbers are shown as pie charts. The size of each chart indicates the number of individuals sampled, which ranged between 5 and 8 individuals, in each location.

Among Palearctic species, the lesser white-toothed shrew (*C. suaveolens*) is one of the most widely distributed shrews in Eurasia that presents the characteristic 2n = 40 chromosomal form [20,21]. Chromosomal polymorphisms, mainly involving pericentric inversions and heterochromatin distribution, have been reported as rare for white-toothed shrews [21]. Although continuously distributed from Siberia to Central Europe [22], the lesser white-toothed shrew is less common and has a more fragmented distribution in Western Europe, including the Iberian Peninsula [23]. In the southwesternmost limit of its distributional range, in the Gulf of Cádiz, *C. suaveolens* is rare, and only occurs in restricted, isolated populations associated to the tidal marches of five rivers (Guadiana, Piedras, Odiel, Tinto and Guadalquivir) [24] (see Figure 1). It has been proposed that its restricted distribution, the strict tidal marsh association and partly the genetic isolation of the populations, could be the consequence of competitive exclusion by the greater white-toothed shrew (*C. russula*, 2n = 42) [25,26]. In fact, recent studies based on mtDNA revealed the presence of two differentiated sub-lineages in Southwestern Iberia that diverged from other Iberian lineages around 140 Ka (50–240 Ka), and between themselves around 110 Ka (30–190 Ka). One of these sub-lineages occupies four river mouths closely located to one another (from 1 to 12 km), in the province of Huelva (Guadiana, Piedras, Odiel and Tinto; sub-lineage C3 [25]), whereas a second sub-lineage was present in the Guadalquivir river mouth (sub-lineage C4 [25]). Subsequent studies using microsatellites revealed that *C. suaveolens* populations from Guadiana, Piedras, Odiel and Tinto rivers showed low differentiation among them, but high differentiation with both the distant Guadalquivir population and the closely located Estero Domingo Rubio (EDR) population [24]. Overall, genetic data suggest that the observed genetic patterns are the result of both historical isolation and current restrictions to gene flow imposed by an adverse landscape matrix [24]. How chromosomal reorganizations have contributed (if so) to this genetic diverge is currently unknown.

Despite their relevance, chromosomal data has often been neglected in phylogeographical studies (reviewed in [27]). The particular distributional range of the habitat-specialist *C. suaveolens* in Southwestern Iberia provides a unique opportunity to investigate whether the formation of genetically isolated populations and habitat specialization was accompanied by chromosomal differentiation. Given this context, in the present study, we aimed to do the following: (i) identify the extent of chromosomal variation *C. suaveolens* populations in Southwestern Iberia, and (ii) determine how chromosomal variation is distributed within and among genetic and phylogeographic groups. In this paper, we discuss the relative contribution of genetic drift and natural selection and the potential evolutionary significance of observed chromosomal differences.

## 2. Materials and Methods

### 2.1. Sampling

A total of 41 individuals from six different populations across the Gulf of Cádiz were collected in 2015 and 2016. This included different localities along the banks of the Guadalquivir, Guadiana, Odiel, Piedras and Tinto rivers (see Figure 1 and Table 1). The sample size ranged from two to six individuals per population. When possible, samples were obtained from both river banks, in order to evaluate whether rivers were acting as barriers to gene flow. Two additional individuals from the Northwest Iberian lineage (lineage B [24]) were also included in the study, for comparison (individuals from the Cáceres and Zamora populations, Table 1).

Tissue samples (ear and tail) were obtained from each specimen on site and transferred to the cell culture laboratory in transport medium (Dulbecco’s Modified Eagle Medium—DMEM— supplemented with 2 mm L-glutamine, 10% fetal bovine serum, 1% 100× Penicillin/Streptomycin/Amphotericin B solution and 0.07 mg/mL Gentamicin). Animals were immediately released after sample collection. Captures were performed with official permits issued by the corresponding nature conservation institutions, and research was conducted with approval of the bioethics committee of the University of Huelva and Universitat Autònoma de Barcelona.

### 2.2. Cell Culture and Chromosomal Harvest

Tissue samples were mechanically and enzymatically disaggregated. Briefly, tissue was washed with 5 mL of DPBS (Dulbecco’s Phosphate-Buffered Saline) solution (DPBS with 1% 100x Penicillin/Streptomycin/Amphotericin B solution and 1mg/mL of Gentamicin) for 10 min, at 37 °C, in an orbital shaker, at 200 rpm. Biopsies were then shredded into small pieces, using a scalpel, in a Petri dish with 1 mL of DMEM without supplements, and incubated for 45 min in DMEM with 0.25% Collagenase Type II at 37 °C and at 200 rpm. Cell suspension was then centrifuged for 10 min at 300 G. The reaming cells were resuspended in 5 mL of completed growth medium (DMEM, supplemented with 20% of fetal bovine serum, 2 mm L-Glutamine) seeded on 25 cm^2^ T-flasks and cultured at standard conditions (37 °C, 10% CO_2_) for four weeks. Cultivated cells proliferated as an adherent monolayer. Subcultures of the adherent cells at early passages (3rd and 4th) were used to obtain chromosomes.

Chromosomal harvest was conducted as previously described [28]. In order to enhance the dispersion of chromosomes, cells were incubated in a hypotonic solution (KCl 0.075 M) for 20 min at 37 °C, inverting every 5 min. Subsequently, the cells were centrifuged (5 min at 300 g) and transferred into 15 mL tubes. The cell pellet was washed twice by adding 5 mL of fixative solution (methanol, acetic acid at 3:1 concentration, freshly prepared) and centrifuged (5 min at 300 g). Cells were centrifuged again and diluted in 1 mL of fixative solution and stored at −20 °C until use.

### 2.3. Chromosomal Characterization

Chromosomal spreads were obtained by dropping 15 µL of cell suspension onto a clean dry slide. Slides were baked at 65 °C during one hour and kept at −20 °C until use. Metaphases were stained homogenously with Giemsa solution for the analysis of the modal karyotype and then G-banded for karyotyping, as previously described [28].

An optical microscope (model Zeiss Axioskop) equipped with a charged coupled device camera (ProgResR CS10Plus, Jenoptik Optical Systems, Jena, Germany) was used for the microscope analysis. A minimum of 25 good-quality metaphases were captured per specimen with the program Progress Capture 2.7.7 and analyzed in order to obtain the modal karyotype. In order to construct representative karyotype of each specimen analyzed, chromosomes were ordered by morphology and decreasing size, resulting in a representative karyotype.

### 2.4. Karyotype Distribution across Lineages and Populations

Several mitochondrial lineages in Iberia were defined previously by Biedma and collaborators [25], two of them (sub-lineages C3 and C4) are present in the study area. Populations were defined as genetic clusters, with each corresponding to one of the disjunct marshes associated to each of the five main rivers in the region (Guadalquivir, Tinto, Odiel, Guadiana and Piedras), and a sixth genetically differentiated population in EDR. Samples were grouped by mitochondrial lineage or geographical population for population cytogenetic analyses.

For the analysis of cytogenetic diversity and differentiation, the A diploid number (autosomes and sex chromosomes) and the presence/absence of B (supernumerary) chromosomes were treated as separate diploid and haploid traits, respectively. GENEPOP on the web (https://genepop.curtin.edu.au; [29]) was used to estimate allele (karyotype) frequencies, observed and expected heterozygosities, and to test for departure from Hardy–Weinberg (HW) expectations. We also tested for genotypic linkage disequilibrium between the two traits (A-chromosomal number and presence/absence of B-chromosomes), using the log likelihood ratio statistics. Differentiation between populations was assessed by the exact G or Fisher’s tests and by estimating Wright’s FST index.

## 3. Results

### 3.1. Chromosomal Diversity in C. suaveolens in the Gulf of Cádiz

**Figure 2:**
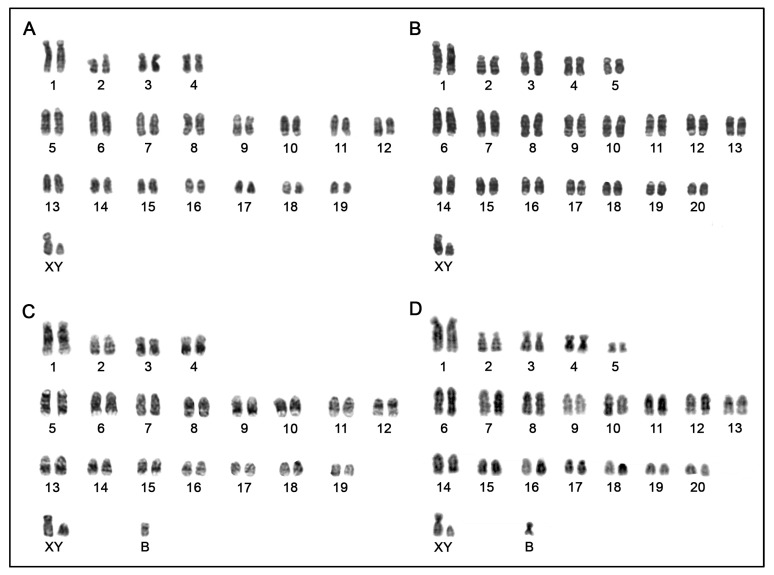
Examples of G-banded karyotypes of *C. suaveolens* found in the studied area. (**A**) Individual from the Guadalquivir River population (2n = 40). (**B**) Individual from the EDR population (2n = 42). (**C**) Individual from the Odiel River population (2n = 41). (**D**) Individual from the Tinto River population (2n = 43). Note the presence of a single B-chromosome in (**C**) and (**D**) karyotypes.

Four karyotype variants were detected in the populations sampled: 2n = 40, 2n = 41 (i.e., 2n = 40 + B), 2n = 42 and 2n = 43 (i.e., 2n = 40 + B; Table 1). All nine individuals from the banks of the Guadalquivir River (sub-lineage C4) were characterized by presenting 2n = 40 (autosomal fundamental number, FNa = 46) (see Figure 2A), the same pattern found in the two individuals from the Northwest Iberian lineage (Cáceres and Zamora). The autosomes consisted of 15 pairs of acrocentric chromosomes and four pairs of bi-armed chromosomes, of which one pair was metacentric and three pairs sub-metacentric. The X-chromosome was a large sub-metacentric (see Figure 2A).

Specimens from three populations (Guadiana, Piedras and EDR) from sub-lineage C3 presented the same karyotype, consisting of 2n = 42 (FNa = 50) (see Figure 2B), with no polymorphic karyomorphs among the 18 individuals analyzed (see Table 1). The autosomes were 15 pairs of acrocentric chromosomes and five pairs of bi-armed chromosomes, of which one pair was metacentric and four pairs were sub-metacentric. The X-chromosome was a large sub-metacentric (see Figure 2B).

Interestingly, chromosomal polymorphisms were detected in both Odiel and Tinto river banks (Table 1), both populations also belonging to sub-lineage C3. In the case of the Odiel population, two distinct karyotypes were found in different proportion: 2n = 42 and 2n = 41. Three out of eight (37.5%) specimens presented the same 2n = 42 karyotype found in Guadiana, Piedras and EDR, whereas five individuals (62.5%) presented a karyotype consisting of 2n = 41 (FNa = 48) chromosomes (see Figure 2C). The 2n = 42 karyotype (FNa = 50) corresponded with the same one found in Guadiana, Piedras and EDR, and it was characterized by the presence of 15 pairs of acrocentric chromosomes and four pairs of bi-armed chromosomes, of which one pair was metacentric and three pairs were sub-metacentric. The 2n = 41 karyotype, however, corresponded to 15 pairs of acrocentric chromosomes and four pairs of bi-armed chromosomes, of which one pair was metacentric and three pairs were sub-metacentric, and the presence of one single B-chromosome was in all the individuals. Since the main difference with the 2n = 40 karyotype found in the Guadalquivir River was the presence of a single B-chromosome, we refer to the 2n = 41 karyotype from Odiel as 2n = 40 + B.

We also found two distinct karyotypes in the Tinto River population. Two out of six (33.3%) individuals surveyed in Tinto presented 2n = 43 (FNa = 52), whereas the rest (66.6%) were characterized by 2n = 42 (the same karyotype formula found in Guadiana, Piedras and EDR). The 2n = 43 karyotype was characterized by 15 pairs of acrocentric chromosomes and five pairs of bi-armed chromosomes, of which one pair was metacentric and four pairs were sub-metacentric, and the presence of a B-chromosome. The X-chromosome was a large sub-metacentric (see Figure 2D). Due to chromosomal G-banding homologies and the presence of a single B-chromosome, we refer to the 2n = 43 karyotype from Tinto as 2n = 42 + B.

G-banding comparison between 2n = 40 and 2n = 42 karyotypes suggests the presence of chromosomal fusion/fission events between bi-armed chromosomes.

### 3.2. Karyotype Distribution and Diversity

**Figure 3:**
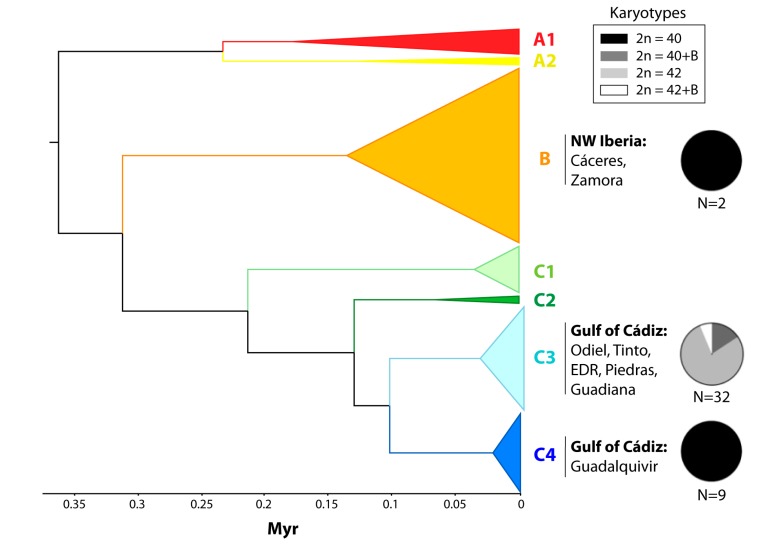
Distribution of karyotypes across the mitochondrial lineages described in Biedma et al. (2019). The geographical distribution of the sub-lineages sampled in this study is indicated. Pie charts represent the frequencies of karyotypes in each mitochondrial lineage, with N referring to the number of individuals sampled.

The four karyotypes observed were unevenly distributed across lineages and populations in the study area. The 2n = 40 A karyotype was the only one observed in the Guadalquivir population, where sub-lineage C4 occurs, and in Zamora and Cáceres samples, which are representative of the B lineage. In contrast, the 2n = 42 A karyotype was the most frequent in C3 populations, and the only one detected in EDR, Piedras and Guadiana (see Figure 3). The presence of B-chromosomes was restricted to the C3 lineage populations, where it occurred in the context of both 2n = 40 (five out of five occurrences) and 2n = 42 A karyotypes (two out of six occurrences). As a result, chromosomal differentiation between mtDNA subclades across both traits was highly significant (Fisher’s exact test, Chi-Squared > 26.46, d.f. = 4, *p* < 0.00002), but it was high and significant for A-chromosome number (F_ST_ = 0.763, N = 82, *p* < 0.00001) and low and not significant for B-chromosome frequencies (F_ST_ = 0.090, N = 41, *p* = 0.179).

Most of the chromosomal diversity in the region is distributed among populations, as most populations showed no karyotypic diversity. Notable exceptions are the neighboring populations of Odiel and Tinto, each showing two composite karyotypes (2n = 40 + B and 2n = 42 in Odiel; 2n = 42 and 2n = 42 + B in Tinto). When only diploid A-chromosomal number is considered, the only population showing both 2n = 40 and 2n = 42 A-complements is Odiel (K_R_ = 2; H_E_ = 0.47; Table 2); although our limited sample sizes do not allow us to exclude the occurrence of both chromosomal types in other populations, results suggest highly unbalanced frequencies if this is the case. Departure from Hardy–Weinberg expectations was significant in Odiel (F_IS_ = 1, N = 8, p = 0.0074) due to the absence of heterokaryotypes despite rather equalized frequencies of the two karyotypes, although we cannot discard a spurious result due to small sample size. Odiel and Tinto were also variable with respect to the presence/absence of B-chromosomes (H = 0.47 and H = 0.44, respectively). The presence of B-chromosome appeared not completely independent of A-chromosome number in Odiel, as B-chromosome was observed there only on a 2n = 40 background (Chi-Squared = 8.02, d.f. = 2, *p* = 0.018).

**Figure 4:**
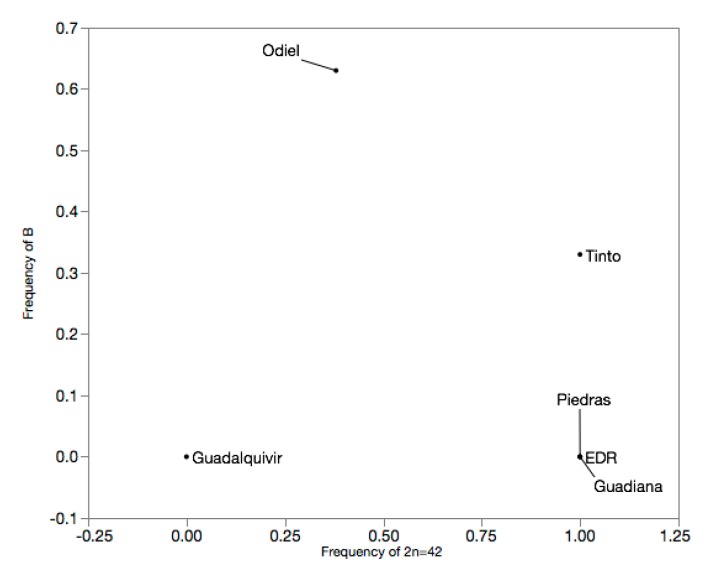
Separation of populations according to frequency of 2n = 42 and B-chromosome.

Overall, karyotypic differentiation among populations was extremely high (Exact G test, N = 41, *p* < 3.98×10^−9^), both for A-chromosome number (F_ST_ = 0.796, N = 41, *p* < 0.0001) and B-chromosome (F_ST_ = 0.408, N = 41, *p* = 0.0014; Figure 4 chromosome (F_ST_ = 0.408, N = 41, *p* = 0.0014; Figure 4).

Karyotypic differentiation for A-chromosome number was maximal (F_ST_ = 1, N = 14–17, p < 0.0001) between Guadalquivir and all other populations, except Odiel (F_ST_ = 0.308, N = 17, *p* = 0.0062), and high between Odiel and Tinto, EDR, Piedras or Guadiana (F_ST_ = 0.496–0.550, N = 13–15, *p* = 0.0014–0.0004; Table 3). Slightly different patterns were observed for B-chromosome, with highest differentiation between Guadalquivir and Odiel (F_ST_ = 0.591, N= 17, *p* = 0.0093) and lowest between Odiel and Tinto (F_ST_ = 0.013, N = 14, *p* = 0.591; Table 3).

## 4. Discussion

### 4.1. Overview of Chromosomal Evolution in Crocidura

Understanding how genomes are organized and which types of chromosomal rearrangements are implicated in macroevolutionary events are fundamental to understanding the dynamics and emergence of new species [30]. Because Crocidura is the largest and one of the most karyotypically diverse genera of the family Soricidae [19,31], it offers a unique opportunity to test the role of chromosomal reorganization in species’ diversification and habitat-specialization.

It has been long assumed that the widespread and monophyletic Palearctic *C. suaveolens* group is characterized by a karyotype of 2n = 40 [15,16]. Exceptions to this rule were initially reported in isolated populations from the Czech Republic (2n = 41) and Switzerland (2n = 42) [21]. Here we extend these initial observations and describe the presence of previously unreported chromosomal variation in the southwesternmost limit of its distributional range, in the Gulf of Cádiz, emphasizing the uniqueness of these genetically isolated and marsh-specialist populations. Karyotypic variability was reflected by the presence of four different karyotypes (2n = 40, 2n = 40 + B, 2n = 42 and 2n = 42 + B), the latter (2n = 42 + B) being reported here for the first time for C. suaveolens. As 2n = 40 is considered the chromosomal form ancestral for *C. suaveolens*, the G-banding comparisons between karyotypes suggest that chromosomal fissions and/or inversions changes in centromeric position together with the emergence of supernumerary (B) chromosomes have originated the chromosomal variability detected in our study. Most notably, our results provide a rare example of a true intrapopulation chromosomal polymorphism in *C. suaveolens*.

Remarkably, two of the karyotypes detected (2n = 40+B and 2n = 42+B) were characterized by the presence of B-chromosomes. Although previous studies recorded the occurrence of B-chromosomes for *C. crossei* [17], *C.* cf. *malayana* [32], *C. poensis* [33] and *C. suaveolens* itself [21], the present study is the first report focusing on the southwesternmost limit of the distributional range of *C. suaveolens*. Despite their widespread distribution in wild populations of several animal, plant and fungi species, the evolutionary origin and function of B-chromosomes are largely unknown. These dispensable chromosomes present a particular behavior, thus not following Mendelian segregation laws [34]. Most B-chromosomes are mainly or entirely heterochromatic (i.e., largely non-coding), although in some cases, B-chromosomes can provide some positive adaptive advantage, as suggested by associations with particular habitats [35] or with increases of crossing over and recombination frequencies [36,37,38]. Interestingly, the presence of B-chromosomes in our study was associated to the C3 mt DNA clade, more particularly in Tinto and Odiel populations. This, together with its generalized rarity in the rest of the distribution, suggests a recent and derivative origin of supernumerary chromosomes. Pending further functional and genomic studies, we can only speculate on the evolutionary implications of B-chromosomes in the *C. suaveolens* populations of Southwestern Iberia.

### 4.2. Karyotypic Diversity and Differentiation of C. suaveolens Populations in the Gulf of Cádiz

The karyotypic diversity detected in the *C. suaveolens* populations in the Gulf of Cadiz reveals the potential of chromosomal differentiation in the isolation of genetically distinct populations of the marsh-specialist *C. suaveolens* (Mammalia: Soricidae). In fact, we found an association between the two differentiated mtDNA sub-lineages (C3 and C4) and diploid numbers. All specimens from the Guadalquivir River’s mouth (sub-lineage C4) were characterized by the ancestral chromosomal form for *C. suaveolens* (2n = 40), which was also present in the individuals sampled from the Northwest Iberian lineage (lineage B [24]). Remarkably, this ancestral karyotype was not detected in sub-lineage C3 populations (i.e., Guadiana, Odiel, Piedras, Tinto and EDR), which presented diploid numbers ranging from 2n = 41 to 2n = 43. Since it has been suggested that both mtDNA sub-lineages’ ages diverged around 110 Ka (30–190 Ka) [25], the relationship between mtDNA divergence and chromosomal reorganizations adds support for the long-term isolation among C3 and C4 lineages.

Within populations of the C3 sub-lineage, the chromosomal form 2n = 42 was the most widespread, suggesting a common (and recent) origin in this lineage, most probably derived by a chromosomal fission from the ancestral form 2n = 40. The presence of 2n = 40 in these populations (always observed in combination with B-chromosome) is thus more parsimoniously explained as the retention of ancestral variation, especially since secondary contact with 2n = 40 populations in the Guadalquivir River is considered unlikely [24,25]. Chromosomal variation in Odiel and Tinto populations may thus constitute a transient “floating” polymorphism, as defined by [27], whose persistence may have been favored by different factors, such as its relatively recent origin (divergence of C3 sub-linage dated ca. 110 ka, [24]), relatively high population sizes and by the spatial structure within the Odiel–Tinto march complex. On the other hand, lack of detected polymorphisms in westernmost populations may be the consequence of a gradual diversity loss during westward colonization of more recent marshes, due to serial founder events [25].

Although chromosomal rearrangements have traditionally been considered to have a strong underdominance effect (e.g., [39]), there is increasing evidence to suggest that the small effect of certain types of rearrangements would favor their persistence within populations as polymorphisms [12]. This is the case, for example, of centric fusions/fissions (e.g., the physical joining of two acrocentric chromosomes by their centromeric regions and vice versa). Chromosomal fusions are particularly extended in nature (reviewed in [27]), occurring in as diverse taxa as mammals, reptiles, insects or mollusks [40,41]. Weak or null selection against centric fusions/fissions heterokaryotes due to mild meiotic pairing dysgenesis or reduction in meiotic recombination also might favor their presence in many lineages, as it has been previously suggested for Primates [42,43,44], Cetartiodactyla [45], the house mouse [12,46] and shrews [47]. Although the limited sample size included in our study calls for caution, the lack of observations of heterokaryotes, despite balanced frequencies of the two karyotypes detected in Odiel, could indicate underdominance in the case of *C. suaveolens* populations, a possibility that deserves further research.

## 5. Conclusions

Our observations for *C. suaveolens* provide an example of the persistence of chromosomal polymorphisms in mammals. The concurrence of chromosomal variation, recently diverged mitochondrial sub-lineages and habitat specialization in *C. suaveolens* populations in the Gulf of Cádiz provides a promising scenario to test the evolutionary significance of chromosomal variation in mammals and assess its contribution to phenotypic and ecological divergence. Future work should address the currently unresolved questions on the possible fitness differences among karyotypes, their role on postzygotic reproductive isolation and their association with morphological or ecological variation.

## Figures and Tables

**Table 1:** Karyotype variants of *Crocidura suaveolens* found in the study area. Information includes population, region, locality, number of specimens (N), diploid chromosomal number (2n), autosomal fundamental number (FNa) and the presence of B-chromosomes (B-chr).

Population	Region	Locality	N	2n	FNa	B-chr
1	Guadalquivir	Eastern bank	4	2n = 40	46	0
1	Guadalquivir	Western bank	3	2n = 40	46	0
1	Guadalquivir	Faginao—Western bank	2	2n = 40	46	0
2	Guadiana	Isla de San Bruno	3	2n = 42	50	0
2	Guadiana	Salinas del Duque	4	2n = 42	50	0
3	Odiel	Cascajera	4	2n = 41/42	48/50	1
3	Odiel	Puntales	4	2n = 41/42	48/50	1
4	Piedras	Salinas	2	2n = 42	50	0
4	Piedras	El Terrón	3	2n = 42	50	0
5	Tinto	Eastern bank (Fosfoyesos)	6	2n = 42/43	48/52	1
6	EDR	Carabelas dock	6	2n = 42	50	0
7	Zamora	Flechas	1	2n = 40	46	0
8	Cáceres	Garganta de la Olla	1	2n = 40	46	0

**Table 2:** Distribution of karyotypes across populations and karyotypic diversity. The frequency distribution of karyotypes for the composite karyotype and the frequency and diversity statistics for the A-chromosome number and B-chromosome presence are shown for each population, mtDNA lineage and for the pool of samples from the Gulf of Cádiz. Diversity is measured as expected heterozygosity and haplotype diversity for A-chromosome number and B-chromosome, respectively.

			Karyotype Frequency				A-Chromosome Number		B- Chromosome	
Population	N	mtDNA Clade	40	40+B	42	42+B	42 freq	HE	B freq	H
*Guadalquivir*	9	C4	1.00	0.00	0.00	0.00	0.00	0.00	0.00	0.00
*Odiel*	8	C3	0.00	0.63	0.38	0.00	0.38	0.47	0.63	0.47
*Tinto*	6	C3	0.00	0.00	0.67	0.33	1.00	0.00	0.33	0.44
*EDR*	6	C3	0.00	0.00	1.00	0.00	1.00	0.00	0.00	0.00
*Piedras*	5	C3	0.00	0.00	1.00	0.00	1.00	0.00	0.00	0.00
*Guadiana*	7	C3	0.00	0.00	1.00	0.00	1.00	0.00	0.00	0.00
*Clade C4*	9		1.00	0.00	0.00	0.00	0.00	0.00	0.00	0.00
*Clade C3*	32		0.00	0.16	0.78	0.06	0.84	0.00	0.22	0.00
*Overall*	41		0.11	0.06	0.30	0.02	0.66	0.45	0.17	0.28

**Table 3:** Karyotypic differentiation among pairs of populations. F_ST_ values are shown for A-chromosomal number (above the diagonal) and B-chromosome (below the diagonal). Asterisks indicate the significance of exact G tests (* *p* < 0.01; ** *p* < 0.001). Pairwise comparisons indicated by “-” could not be estimated due to lack of variation in the pair.

	mtDNA Clade					
	C4	C3				
	*Guadalquivir*	*Odiel*	*Tinto*	*EDR*	*Piedras*	*Guadiana*
*Guadalquivir*		0.308 *	1000 **	1000 **	1000 **	1000 **
*Odiel*	0.591 *		0.525 **	0.525 **	0.496 *	0.550 **
*Tinto*	0.287	0.013		-	-	-
*EDR*	-	0.525	0.200		-	-
*Piedras*	-	0.496	0.161	-		-
*Guadiana*	-	0.550	0.233	-	-	

## References

[1] Faria R., Johannesson K., Butlin R.K., Westram A.M. (2019). Evolving Inversions. Trends Ecol. Evol..

[2] Hoffmann A.A., Rieseberg L.H. (2008). Revisiting the impact of inversions in evolution: From population genetic markers to drivers of adaptive shifts and speciation?. Annu. Rev. Ecol. Evol. Syst..

[3] Kapun M., Flatt T. (2019). The adaptive significance of chromosomal inversion polymorphisms in *Drosophila melanogaster*. Mol. Ecol..

[4] Ayala D., Zhang S., Chateau M., Fouet C., Morlais I., Costantini C., Hahn M.W., Besansky N.J. (2019). Association mapping desiccation resistance within chromosomal inversions in the African malaria vector *Anopheles gambiae*. Mol. Ecol..

[5] Christmas M.J., Wallberg A., Bunikis I., Olsson A., Wallerman O., Webster M.T. (2019). Chromosomal inversions associated with environmental adaptation in honeybees. Mol. Ecol..

[6] Gündüz I., Auffray J.C., Britton-Davidian J., Catalan J., Ganem G., Ramalhinho M.G., Mathias M.L., Searle J.B. (2001). Molecular studies on the colonization of the Madeiran archipelago by house mice. Mol. Ecol..

[7] Dumas D., Britton-Davidian J. (2002). Chromosomal rearrangements and evolution of recombination: Comparison of chiasma distribution patterns in standard and Robertsonian populations of the house mouse. Genetics..

[8] Giménez M.D., Mirol P.M., Bidau C.J., Searle J.B. (2002). Molecular analysis of populations of *Ctenomys.* (Caviomorpha, Rodentia) with high karyotypic variability. Cytogenet. Genome Res..

[9] Borodin P.M., Karamysheva T.V., Belonogova N.M., Torgasheva A.A., Rubtsov N.B., Searle J.B. (2008). Recombination map of the common shrew, *Sorex araneus.* (Eulipotyphla, Mammalia). Genetics.

[10] Franchini P., Colangelo P., Solano E., Capanna E., Verheyen E., Castiglia R. (2010). Reduced gene flow at pericentromeric loci in a hybrid zone involving chromosomal races of the house mouse *Mus musculus domesticus*. Evolution.

[11] Förster D.W., Mathias M.L., Britton-Davidian J., Searle J.B. (2013). Origin of the chromosomal radiation of Madeiran house mice: A microsatellite analysis of metacentric chromosomes. Heredity.

[12] Capilla L., Medarde N., Alemany-Schmidt A., Oliver-Bonet M., Ventura J., Ruiz-Herrera A. (2014). Genetic recombination variation in wild Robertsonian mice: On the role of chromosomal fusions and Prdm9 allelic background. Proc. R. Soc. B Biol. Sci..

[13] Vara C., Capilla L., Ferretti L., Ledda A., Sánchez-Guillén R.A., Gabriel S.I., Albert-Lizandra G., Florit-Sabater B., Bello-Rodríguez J., Ventura J. (2019). PRDM9 Diversity at fine geographical scale reveals contrasting evolutionary patterns and functional constraints in natural populations of house mice. Mol. Biol. Evol..

[14] O’Brien S.J., Menninger J.C., Nash W.G. (2006). An Atlas of Mammalian Chromosomes.

[15] Dubey S., Salamin N., Ohdachi S.D., Barrière P., Vogel P. (2007). Molecular phylogenetics of shrews (Mammalia: Soricidae) reveal timing of transcontinental colonizations. Mol. Phylogenet. Evol..

[16] Dubey S., Salamin N., Ruedi M., Barrière P., Colyn M., Vogel P. (2008). Biogeographic origin and radiation of the Old World crocidurine shrews (Mammalia: Soricidae) inferred from mitochondrial and nuclear genes. Mol. Phylogenet. Evol..

[17] Maddalena T., Ruedi M., Merrit J.F., Kirkland G.L., Rose R.K. (1994). Chromosomal Evolution in the Genus Crocidura. (Soricidae, Insectivora). Advances in the Biology of Shrews.

[18] Castiglia R., Annesi F., Sichilima A.M., Hutterer R. (2009). A molecular and chromosomal study of the moonshine shrew, *Crocidura luna Dollman*, 1910 from Zambia with a description of a new remarkable karyotype. Mammalia.

[19] Biltueva L., Vorobieva N., Perelman P., Trifonov V., Volobouev V., Panov V., Ilyashenko V., Onischenko S., O’Brien P., Yang F. (2001). Karyotype evolution of eulipotyphla (insectivora): The genome homology of seven sorex species revealed by comparative chromosome painting and banding data. Cytogenet. Genome Res..

[20] Meylan A., Hausser J. (1974). Cytotaxonomic position of several shrews of the genus Crocidura in Tessin (Mammalia, Insectivora). Rev. Suisse Zool..

[21] Zima J., Lukacova L., Machola M., Wojcik J.M., Wolsan M. (1998). Chromosomal evolution in shrews. Evolution of Shrews.

[22] Palomo L., Kryštufek B., Amori G., Hutterer R. *Crocidura suaveolens*. The IUCN Red List of Threatened Species 2016: E.T29656A22296429. http://www.iucnredlist.org/details/29656/.

[23] Libois R., Ramalhinho M.G., Fons R., Mitchell-Jones A.J., Amori G., Bogdanowicz W., Kryštufek B., Reijnders P.J.H., Spitzenberger F., Stubbe M., Thissen J.B.M., Vohralik V., Zima J. (1999). Crocidura. suaveolens. (Pallas, 1811), the lesser-white toothed shrew. The Atlas of European Mammals.

[24] Biedma L., Calzada J., Román J., Godoy J.A. (2019). Rare and rear: Population genetics of marsh-specialist *Crocidura. suaveolens.* populations in the Gulf of Cádiz. J. Mammal..

[25] Biedma L., Román J., Calzada J., Friis G., Godoy J.A. (2018). Phylogeography of *Crocidura suaveolens* (Mammalia: Soricidae) in Iberia has been shaped by competitive exclusion by *C. russula*. Biol. J. Linn. Soc..

[26] Biedma L., Calzada J., Godoy J.A., Román J. (2020). Local habitat specialization as an evolutionary response to interspecific competition between two sympatric shrews. J. Mammal..

[27] Dobigny G., Britton-Davidian J., Robinson T.J. (2004). Chromosomal polymorphism in mammals: An evolutionary perspective. Biol. Rev..

[28] Ruiz-Herrera A., Ponsà M., García F., Egozcue J., García M. (2002). Fragile sites in human and *Macaca fascicularis* chromosomes are breakpoints in chromosome evolution. Chromosome Res..

[29] Raymond M., Rousset F. (1995). GENEPOP (Version 1.2): Population genetics software for exact tests and ecumenicism. Heredity.

[30] Ruiz-Herrera A., Farré M., Robinson T.J. (2012). Molecular cytogenetic and genomic insights into chromosomal evolution. Heredity.

[31] Hutterer R., Wilson D.E., Reeder D.M. (2005). Order Soricomorpha. Mammal Species of the World: A Taxonomic and Geo-Graphic Reference.

[32] Ruedi M., Vogel P. (1995). Chromosomal evolution and zoogeographical origin of southeast Asian shrews (genus *Crocidura*). Experientia.

[33] Maddalena T., Peters G., Hutterer R. (1990). Systematics and biogeography of Afrotropical and Paleartic hrews of the genus *Crocidura* (Insectivora: Soricidae): An electrophoretic approach. Vertebrates in the Tropics.

[34] Vujosevic M., Rajičić M., Blagojević J.B. (2018). Chromosomes in Populations of Mammals Revisited. Genes.

[35] Goodwin S., M’barek S.B., Dhillon B., Wittenberg A.H., Crane C.F., Hane J.K., Foster A.J., Van der Lee T.A., Grimwood J., Aerts A. (2011). Finished genome of the fungal wheat pathogen *Mycosphaerella graminicola* reveals dispensome structure, chromosome plasticity, and stealth pathogenesis. PLoS Genet..

[36] Patton J.L. (1972). A complex system of chromosomal variation in the pocket mouse, *Perognathus baileyi Merriam*. Chromosoma.

[37] Dvorak J., Deal K.R., Luo M.C. (2006). Discovery and mapping of wheat Ph1 suppressors. Genetics.

[38] Thomson R.L. (1984). B chromosomes in *Rattus fuscipes* II. The transmission of B chromosomes to offspring and population studies: Support for the “parasitic” model. Heredity.

[39] Baker R.J., Bickham J.W. (1979). Speciation by monobrachial centric fusions. Proc. Natl. Acad. Sci. USA.

[40] White M.J.D. (1973). Animal Cytology and Evolution.

[41] King M. (1993). Species Evolution: The Role of Chromosome Change.

[42] Hamilton A.E., Beuttner-Janusch J., Chu E.H. (1977). Chromosomes of lemuriformes. II. Chromosome polymorphism in Lemur fulvus collaris (E. Geoffroy 1812). Am. J. Phys. Anthropol..

[43] Farré M., Micheletti D., Ruiz-Herrera A. (2013). Recombination rates and genomic shuffling in human and chimpanzee: A new twist in the chromosomal speciation theory. Mol. Biol. Evol..

[44] Ullastres A., Farré M., Capilla L., Ruiz-Herrera A. (2014). Unraveling the effect of genomic structural changes in the rhesus macaque-implications for the adaptive role of inversions. BMC Genom..

[45] Rubes J., Pagacova E., Kopecna O., Kubickova S., Cernohorska H., Vahala J., Di Berardino D. (2007). Karyotype, centric fusion polymorphism and chromosomal aberrations in captive-born mountain reedbuck (*Redunca fulvorufula*). Cytogenet. Genome Res..

[46] Medarde N., López-Fuster M.J., Muñoz-Muñoz F., Ventura J. (2012). Spatio-temporal variation in the structure of a chromosomal polymorphism zone in the house mouse. Heredity.

[47] Qumsiyeh M.B., Coate J.L., Peppers J.A., Kennedy P.K., Kennedy M.L. (1997). Roberstonian chromosomal rearrangements in the short-tailed shrew *Blarina carolinensis*, in Western Tennessee. Cytogenet. Genome Res..

